# Intermittent Brugada Syndrome Presenting with Syncope in an Adult Female

**DOI:** 10.1155/2014/742076

**Published:** 2014-10-14

**Authors:** Patricia Chavez, Daniel Bamira, Abel Casso Dominguez, Akshai Bhandary, Eyal Herzog

**Affiliations:** ^1^Division of Cardiology, St. Luke's-Roosevelt Hospital Center, Mount Sinai Health System, New York, NY 10025, USA; ^2^Department of Medicine, St. Luke's-Roosevelt Hospital Center, Mount Sinai Health System, New York, NY 10025, USA

## Abstract

*Background.* Brugada syndrome accounts for 4–12% of all sudden deaths worldwide and at least 20% of sudden deaths in patients with structurally normal hearts. *Case Report.* A 48-year-old female presented to the emergency department after two witnessed syncopal episodes. While awaiting discharge had a third collapse followed by cardiac arrest with shockable rhythm. Initial electrocardiogram showed wide QRS complex with left axis deviation, ST-segment elevation of <1 mm in V1 and V2, and flattening of T waves in V1. The angiogram did not demonstrate obstructive coronary disease. The electrocardiogram obtained two days after these events showed a right bundle branch block with ST-segment elevation of >2 mm followed by a negative T wave with no isoelectric separation, suggestive of spontaneous intermittent Brugada type 1 pattern. Cardiac magnetic resonance imaging demonstrated neither structural heart disease nor abnormal myocardium. After placement of an implantable cardioverter defibrillator the patient was discharged. Why should an emergency physician be aware of this? Brugada syndrome is an infrequently encountered clinical entity which may have a fatal outcome. This syndrome primarily presents with syncope. It should be considered as a component of differential diagnosis in patients with family history of syncope and sudden cardiac death.

## 1. Introduction

Brugada syndrome was first described in 1992 and accounts for 4–12% of all sudden deaths worldwide and at least 20% of sudden deaths in patients with structurally normal hearts [1]. The syndrome is characterized by a history of syncope or sudden cardiac death which occurs due to ventricular tachyarrhythmias [2]. The typical electrocardiographic changes are characterized by downsloping ST-segment elevation in the right precordial leads in patients with structurally normal hearts, but these characteristics exhibit day-to-day variation and may not always be present [1, 2]. The key to management is immediate recognition, evaluation, and treatment of Brugada pattern, when appropriate. This is a case of spontaneous occurring intermittent Brugada type 1 pattern complicated by cardiac arrest.

## 2. Case Report

**Figure 1:**
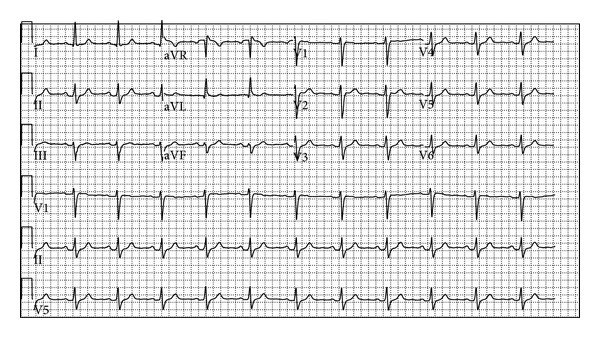
ECG at presentation.

A 48-year-old female with a known history of hypertension, diabetes mellitus, and hypothyroidism was admitted to the emergency department after she experienced two syncope episodes that were accompanied with involuntary jerking movements. It was learnt that she had no prodromal manifestations of dizziness, palpitations, or diaphoresis. While awaiting discharge she had a third witnessed collapse followed by a cardiac arrest in the setting of a shockable rhythm. Her home medications included aspirin and levothyroxine. It was also revealed that these two episodes resolved spontaneously and she had no confusion afterward. Upon arrival to the emergency department she was hemodynamically stable. Of note patient had a son who died at age of 19 years. The initial electrocardiogram (ECG) obtained in the emergency department is shown (Figure 1).

*Question: Does the Patient Have an Underlying Primary Electrical Disturbance?* This ECG shows normal sinus rhythm with a widened QRS complex (140 ms) at 66 beats per minute with a left axis deviation. On careful inspection, ST-segments in V1 and V2 show a <1 mm elevation and flattening of T waves in V1, in this otherwise normal ECG.

**Figure 2:**
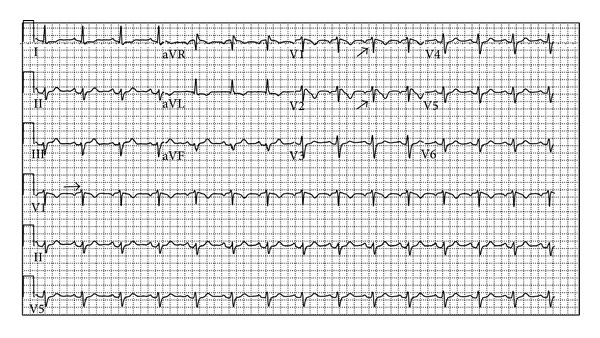
ECG at day 2. Observe the RBBB morphology with the coved-type ST-segment elevation of ≥2 mm followed by a negative T wave.

When the cardiac arrest occurred, the initial rhythm was found to be ventricular fibrillation. The patient received resuscitation per ACLS protocol, including being defibrillated five times in the emergency room with successful return of spontaneous circulation. The angiogram did not demonstrate any obstructive coronary disease. The ECG, obtained two days after these events, is shown (Figure 2). A right bundle branch block (RBBB) with an ST-segment elevation of ≥2 mm followed by a negative T wave with no isoelectric separation and a QTc 440 ms are seen as well as nonspecific T wave changes. These findings suggested a spontaneous intermittent Brugada type 1 pattern. In the majority of the reported cases, there is a coved-type ECG in the right precordial leads before the development of ventricular arrhythmias (2). The patient was found to be febrile at this time. Cardiac MRI was performed and demonstrated neither structural heart disease nor abnormal myocardium. The patient was discharged home after a successful single-lead implantable cardioverter defibrillator (ICD) placement. She remained asymptomatic.

## 3. Discussion

While neurally mediated collapse is the most common cause of syncope, arrhythmias that emerge primarily or secondarily to structural heart disease may also present as syncope. In this case, the absence of a prodrome is consistent with cardiac arrhythmia. Evaluation of syncope should include evaluation of coronary artery disease, nonischemic dilated cardiomyopathy, and other forms of structural heart disease. Inherited cardiac ion channel abnormalities should also be considered, with long QT and Brugada syndromes being the most common. Brugada syndrome is diagnosed when a type 1 ST-segment elevation is observed in at least 1 right precordial lead and in conjunction with one of the following: ventricular fibrillation (VF), polymorphic ventricular tachycardia (VT), a family history of sudden cardiac death at young age (under 45 years old), typical ECG changes in family members, inducible VT with programmed electrical stimulation, syncope, or nocturnal agonal respiration. Brugada syndrome accounts for 4–12% of all sudden deaths worldwide and at least 20% of sudden deaths in patients with structurally normal hearts [1, 2]. In the United States, the prevalence of Brugada syndrome has been indicated as 0.03% which is significantly lower than those of Asia and Europe [3]. The male to female ratio of Brugada syndrome is about 10 : 1 [4]. Genetic mutations at the level of ion channels have been identified, the most common being a mutation in SCN5A, the gene encoding the alpha subunit of the cardiac sodium channel [5]. Other mutations involving potassium and calcium channels have been acknowledged as well [6]. No genetic studies were performed in the aforementioned patient as she had no other known family. We believe the intermittent pattern of Brugada syndrome in our patient was unmasked by fever, which is a known trigger [7]. Intriguingly Brugada syndrome ECG patterns are often seen at night or rest, after large meals, and even with increased vagal tone [6].

Patients with Brugada syndrome who present with syncope have a 2-year risk of sudden cardiac death of approximately 30% [8]. Both familial history of SCD or coved-type ST-segment elevation have not proven to be significant risk factors for future arrhythmic events [9]. Remarkably, changes of ≥0.2 mm in the ST level of the right precordial leads, as in this case, are more frequently observed in the ventricular fibrillation [10]. ICD was appropriately implanted for secondary prevention of sudden death.

*Why Should an Emergency Physician Be Aware of This?* Brugada syndrome is an infrequently encountered clinical entity which may have a possibly fatal outcome. This syndrome primarily presents with syncope. A diagnosis of Brugada syndrome should be considered in patients with typical ECG alterations, personal or family history of syncope and sudden cardiac death, or other criteria that are known to be associated with the disease process.
